# Cyclophilin D ablation is associated with increased end-ischemic mitochondrial hexokinase activity

**DOI:** 10.1038/s41598-017-13096-7

**Published:** 2017-10-06

**Authors:** Rianne Nederlof, Mark A. M. van den Elshout, Anneke Koeman, Laween Uthman, Iris Koning, Otto Eerbeek, Nina C. Weber, Markus W. Hollmann, Coert J. Zuurbier

**Affiliations:** Laboratory of Experimental Intensive Care and Anesthesiology, Department of Anesthesiology, Academic Medical Center, Meibergdreef 9, 1105 AZ Amsterdam, The Netherlands

## Abstract

Both the absence of cyclophilin D (CypD) and the presence of mitochondrial bound hexokinase II (mtHKII) protect the heart against ischemia/reperfusion (I/R) injury. It is unknown whether CypD determines the amount of mtHKII in the heart. We examined whether CypD affects mtHK in normoxic, ischemic and preconditioned isolated mouse hearts. Wild type (WT) and CypD^−/−^ mouse hearts were perfused with glucose only and subjected to 25 min ischemia and reperfusion. At baseline, cytosolic and mtHK was similar between hearts. CypD ablation protected against I/R injury and increased ischemic preconditioning (IPC) effects, without affecting end-ischemic mtHK. When hearts were perfused with glucose, glutamine, pyruvate and lactate, the preparation was more stable and CypD ablation^−^resulted in more protection that was associated with increased mtHK activity, leaving little room for additional protection by IPC. In conclusion, in glucose only-perfused hearts, deletion of CypD is not associated with end-ischemic mitochondrial-HK binding. In contrast, in the physiologically more relevant multiple-substrate perfusion model, deletion of CypD is associated with an increased mtHK activity, possibly explaining the increased protection against I/R injury.

## Introduction

Ischemia and reperfusion cause oxidative stress, elevated phosphate concentrations, adenine nucleotide depletion and calcium overload. This leads to opening of the mitochondrial permeability transition pore (MPTP), a non-specific pore in the inner mitochondrial membrane, which causes cell death^1^. Dimerization of F_0_F_1_ATPase has recently been proposed as the molecular identity of the MPTP with an important regulatory role for cyclophilin D (CypD)^2^. Inhibiting CypD with cyclosporine A (CsA) or a knock-out in the gene coding for CypD, delays opening of the MPTP and commonly reduces ischemia-reperfusion (I/R) injury^3–9^.

Another important mediator of MPTP opening is the glycolytic enzyme hexokinase II (HKII). HKII can be found at two different places in the cell, bound to the mitochondria or free in the cytosol. When bound to the mitochondria, HKII protects against reactive oxygen species or calcium induced pore opening^10^. We have shown that mitochondrial HKII (mtHKII) protects against I/R injury in skeletal and cardiac muscle^11–14^ and that cardioprotective interventions increase mitochondrial hexokinase activity (mtHK) before and after (but not during) the prolonged period of ischemia^15–17^. In addition we have shown that a disruption of the mitochondrial-HK binding blocks ischemic preconditioning^13^. The data indicate that increases in mtHK are necessary for ischemic preconditioning to be effective^13,18,19^.

Previous work has demonstrated that CypD^−/−^ cardiomyocytes and CypD knock-out (KO) mice are protected against I/R injury, but could not be further protected by IPC^5,7^. This raises the question whether CypD and HKII interact in I/R injury and protection thereof in the intact heart. Indeed, an interaction between CypD and mtHKII has been found in cancer cells, albeit in the opposite direction^20^. In addition, inhibiting CypD activity reverted mitochondrial depolarization and prevented cell death caused by a peptide that detaches HKII from mitochondria in fibroblasts^21^. This data indicates a functional link between CypD and HK binding to mitochondria^22^. However, to what extent CypD presence and activity affect mitochondrial hexokinase activity in the intact heart during baseline conditions or during ischemia with or without preceding IPC remains unknown. Therefore, in the present study we examine in the intact mouse heart whether 1) mitochondrial HK association depends on the presence of CypD, 2) CypD effects on I/R injury are mirrored by alterations in end-ischemia mtHK, and 3) the suggested loss of IPC cardioprotection with CypD ablation prevents end-ischemia mtHK increases.

## Material and Methods

### Animals

C57BL/6 J CypD^−/−^ were a generous gift of dr. M. Forte, Oregon Health and Science University, Oregon, USA. This mouse was first described by Basso *et al*.^4^ and has a deletion in the Ppif gene, coding for CypD. A heterozygous CypD^+/−^ mouse was created by crossing CypD^−/−^ males with C57BL/6 J females (Harlan). Subsequently a WT and CypD^−/−^ line were created from these CypD^+/−^ animals. Genotyping was performed by PCR on DNA isolated from toe biopsies of each animal at the start of breeding and later of one animal per nest. Cardiac energetics experiment were performed in 20–25 g male C57BL/6 J mice (Harlan). Mice were housed under standard housing conditions (12 h dark/12 h light cycle; water and food given *ad libitum*). A total of 138 male mice of 10–18 weeks of age were examined. All experiments were approved by the animal ethics committee of the Academic Medical Center, Amsterdam, The Netherlands and performed conform the guidelines from Directive 2010/63/EU of the European Parliament on the protection of animals used for scientific purposes.

### Heart perfusion

Isolated heart perfusion was performed as described previously with slight modifications^23,24^. In brief, mice were heparinized (15 IU) and anaesthetized using pentobarbital (Euthasol 20%, AST Farma) (100 mg/kg intra peritoneal). Adequate level of anaesthesia was determined by the lack of toe pinch reflexes. Tracheotomy was performed and mice were mechanically ventilated. After thoracotomy hearts were cannulated *in situ* and perfusion was started before excision of the heart. Hearts were Langendorff perfused with a constant perfusion flow (initial perfusion pressure of 80 mmHg) at 37 °C with Krebs-Henseleit solution (KHB) containing (mmol/L) NaCl 118, KCl 4.7, CaCl_2_ 2.25, MgSO_4_ 1.2, NaHCO_3_ 25, KH_2_PO_4_ 1.2 and EDTA 0.5 gassed with 95% O_2_/5% CO_2_. Depending on the study group the following substrates were added to the KHB (mmol/L) only glucose 11, or glucose 11, glutamine 0.5, lactate 1.0 and pyruvate 0.1. The perfusate was filtered in-line with a 0.45 μm filter. A water-filled polyethylene balloon was inserted in the left ventricular cavity and end diastolic pressure (EDP) was set at ~4–8 mmHg. Hearts were continuously submerged in 37 °C KHB. During ischemia hearts were submerged in KHB gassed with 95% N_2_/5% CO_2_. Developed left ventricular pressure (DLVP) was calculated as the systolic pressure minus the EDP. Rate pressure product (RPP) was calculated as DLVP * heart rate. Time to onset of contracture (TOC) was determined as the time from which diastolic pressure raised above baseline, followed by a consistent increase^25^.

### Protocol

**Figure 1:**
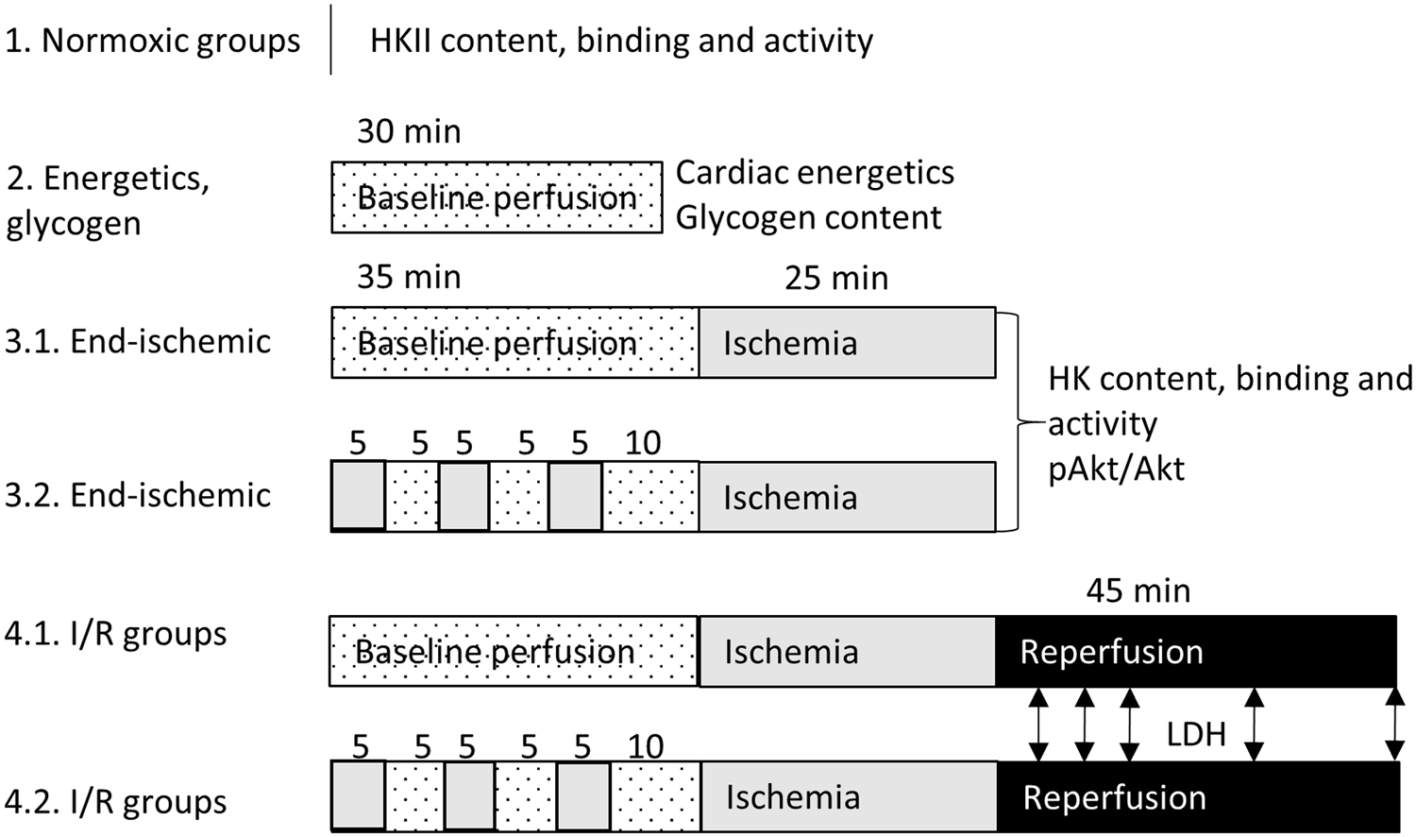
Schematic overview of the different protocols. All hearts were exposed to 20 min perfusion with either glucose only, or multi-substrate perfusate to stabilize the heart. Hearts of group 1 were then homogenized and mitochondria were isolated for determination of mitochondrial hexokinase amount and activity. Hearts of group 2 were exposed to 30 min baseline perfusion after which they were frozen and used for cardiac energetics and glycogen determination. Hearts of group 3 were exposed to 35 min baseline perfusion with or without IPC, followed by 25 min no-flow ischemia. Then hearts were homogenized and mitochondria isolated for determination of HKII amount and activity and Akt phosphorylation. Hearts of group 4 were exposed to 35 min baseline perfusion with or without IPC, 25 min ischemia and 45 min reperfusion. LDH was sampled at 5, 10, 15, 30 and 45 min reperfusion to determine cardiac damage. Hearts of group 5 were exposed to 20 min baseline perfusion with multi-substrate buffer, followed by 25 min ischemia and 10 min reperfusion. White: baseline perfusion with multi-substrate perfusate, white dotted: baseline perfusion with both glucose-only and multi-substrate perfusate, grey: no-flow ischemia, black: reperfusion.

Figure 1 shows a schematic overview of the different perfusion protocols used. Isolated hearts were Langendorff perfused for ~20 min to reach stable conditions after which the hearts were exposed to different protocols.

1. Normoxic groups: WT and CypD^−/−^ hearts (n = 6 per group; 2 groups) were perfused with KHB with glucose only. After stabilisation hearts were weighed and immediately homogenized. These experiments evaluated possible differences in HK between WT and CypD^−/−^ hearts under baseline conditions.
2. Energetics and glycogen group: C57BL/6 J hearts (n = 6–7 per group; 2 groups) were perfused with the two different kinds of KHB. After 30 min basal perfusion hearts were snap frozen in liquid nitrogen, weighed and stored at −80 °C until analysis. This series examined the consequences of the different substrate perfusate mixture on cardiac energetics and glycogen under baseline conditions.
3. End-ischemic groups: WT and CypD^−/−^ hearts (n = 6 per group; 8 groups) were perfused with the two different kinds of KHB. Half of the groups were exposed to IPC, consisting of 3 × 5 min global ischemia interspersed with 5 min reperfusion, with the last reperfusion period lasting 10 min. Control hearts were continuously perfused for 35 min. Thereafter, all hearts were subjected to 25 min ischemia and homogenized to isolate mitochondria. This series studied whether ischemia with or without IPC affects mtHK differently in WT versus CypD^−/−^ hearts. In hearts perfused with glucose, glutamine, lactate and pyruvate, pAkt/Akt was determined in whole homogenate.
4. I/R groups: WT and CypD^−/−^ hearts (n = 6–13 per group; 8 groups) were perfused with the two different kinds of KHB with or without preceding IPC. All hearts were subjected to 25 min ischemia and 45 min reperfusion for determination of I/R injury and IPC protective effects. At the end of reperfusion wet weight was determined for all hearts. This series answered the question to what extent the ablation of CypD affected the cardiac response to I/R injury with or without IPC.

### Mitochondria isolation

For protocols 1 and 3 mitochondria were isolated according to Pasdois *et al*.^19^ with a few modifications. All homogenisation steps were performed at 4 °C. Hearts were homogenized 2 min with a Potter homogenizer at 1200/min in 1.5 mL homogenisation buffer containing (mmol/L) sucrose 250, HEPES 20, KCl 10, MgCl_2_ 1.5, EDTA 1, glucose 5, protease inhibitor (Roche) and phosphatase inhibitors (Roche), pH 7.4. Part of the whole homogenate was stored immediately at −80 °C, the rest of the homogenate was centrifuged 7 min at 7500 g. The resulting supernatant is the cytosolic fraction and was stored at −80 °C. The pellet was resuspended in 1.5 mL homogenisation buffer and further homogenized 3 min at 1200/min. The homogenate was centrifuged 10 min at 700 g and the resulting supernatant was centrifuged 10 min at 7000 g. The crude mitochondrial pellet was resuspended in homogenisation buffer with 25% (w/v) Percoll (pH 7.1 to 7.2 at 4 °C), homogenized for 20 s as described above and centrifuged for 10 min at 17 000 g. The pellet was resuspended in 1.5 mL homogenisation buffer and centrifuged 10 min at 7000 g. The pellet contained the purified mitochondria and was stored at −80 °C until use.

### Western blotting

HKII and Akt were determined using Western blot. HKII in normoxic and end-ischemic cytosolic and mitochondrial samples, Akt in end-ischemic whole homogenate. Western blotting was performed as described before^26–28^. In short, equal amounts whole-cell homogenate, cytosol or mitochondrial protein were loaded and electrophoresed on a 4–12% gradient gel (Biorad) and transferred to a polyvinylidene membrane. The membrane was probed with an antibody for HKII (1:10 000; Cell signalling #2867), phospho-Akt (Ser473) (1:500; Cell Signalling #9271), Akt (1:1000; Cell Signalling #9272) and the cytosolic marker alpha-tubulin (1:40 000; Sigma T9026) or the mitochondrial marker VDAC (1:5000; Calbiochem PC548) or COX IV (1:9000; Cell Signalling 4844). To facilitate comparison among the 4 groups within each substrate-specific perfusion series samples were analysed within the same blot. Protein concentrations of the fractions were determined by the Bradford method.

### Enzyme activity assays

Whole-cell homogenate and the mitochondrial fraction were treated with 0.5% Triton X-100, sonificated 3 × 5 s and centrifuged 1 min at 10 000 g. Enzyme activity was determined in the supernatant of these fractions^26^.

Total lactate dehydrogenase (LDH) activity was determined at 25 °C in whole homogenate of hearts of protocol 1 using a spectrophotometric assay measuring NADH oxidation at 340 nm after addition of pyruvate. LDH activity was corrected for protein concentration.

In addition, LDH release in effluent was used as an index of cardiac necrosis. Other studies have shown a good correlation between LDH release and infarct size determined by TTC staining^29–31^. LDH activity was determined in effluent collected at 5, 10, 15, 30 and 45 min reperfusion. Total LDH release during the 45 min reperfusion was calculated and normalized to total LDH content of normoxic hearts.

Hexokinase activity was determined in whole homogenate, isolated mitochondria and cytosol of protocol 1 and 3. It was measured spectrophotometrically at 25 °C with glucose-6-phosphate dehydrogenase, glucose, ATP and NAD^+^, in the presence of rotenone (1 μM) to inhibit mitochondrial respiration. Hexokinase activity in total homogenate and the cytosolic fraction was corrected for protein concentration, and in the mitochondria for citrate synthase (CS), a mitochondrial marker. CS was measured at 25 °C using acetyl-CoA, oxaloacetate and di-thionitrobenzoic acid, measuring the formation of thionitrobenzoic acid at 412 nm.

### Cardiac energetics and glycogen measurements

Hearts from protocol 2 were freeze-dried overnight and dry weight was determined. In one part of the heart ATP, phosphocreatine (PCr), creatinine (Cr) and inorganic phosphate (Pi) were measured as described by Fiolet *et al*.^32^. The phosphorylation potential (ΔG_ATP_) was calculated from these values. In the other part glycogen content was determined using the glycogen colorimetric/fluorometric assay kit (Biovision) according to manufacturer’s instructions.

### Statistical analysis

Data are presented as mean + SEM, and sample sizes are provided in the legend of the figures. For the I/R outcome parameters of LDH release, EDP and RPP recovery a n = 7 was initially chosen based on previous studies^23^; with this n we could detect a difference of 45% among groups with an α = 0.05 and a power of 0.8 based on an SD of 13. For the HK and Akt parameters n = 6 for all 4 groups was used to be able to make comparisons on one 26 lane gel. Analysis of the important parameters (LDH and HK) was performed blind. Testing was performed using SPSS version 21. Mann-Whitney U tests were performed to test for significance between 1) control treated WT and CypD^−/−^ animals 2) control and IPC treated animals in WT and 3) CypD^−/−^ animals. Glycogen and cardiac energetics data were analysed using an Independent T-test. A p-value < 0.05 was considered significant.

## Results

### Baseline characteristics WT and CypD^−/−^ hearts

**Figure 2:**
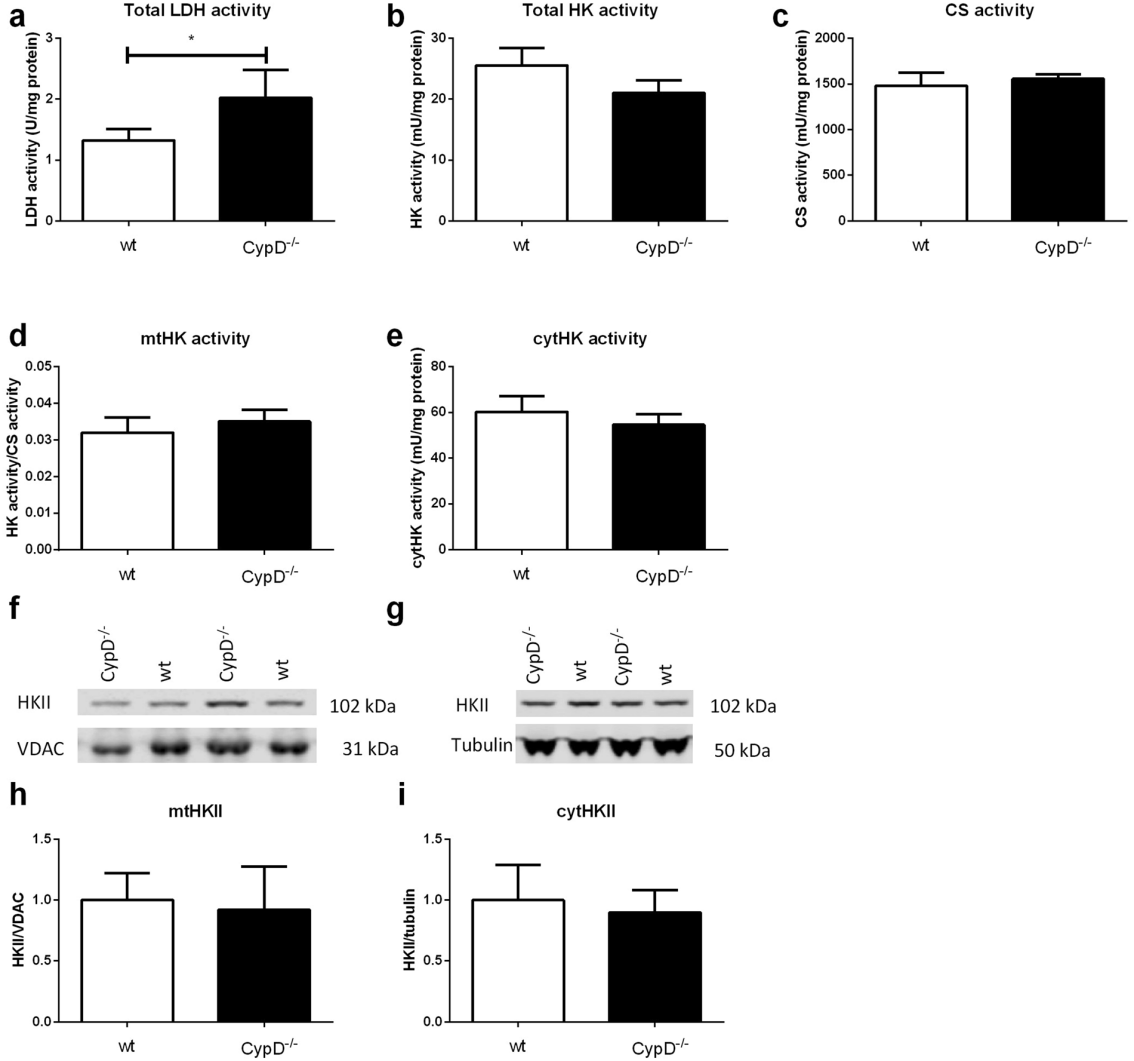
Baseline characteristics of WT and CypD^−/−^ hearts. (**a**) Total lactate dehydrogenase (LDH) activity of CypD^−/−^ hearts normalized to protein content. (**b**) Hexokinase activity in whole heart normalized to protein content. (**c**) Mitochondrial citrate synthase (CS) activity normalized to protein content as parameter of mitochondrial capacity. (**d**) Mitochondrial HK activity as ratio to CS activity. (**e**) Cytosolic HK activity normalized to protein content. (**f**) Representative Western blot images of mitochondrial HKII/VDAC and (**g)** cytosolic HKII/tubulin. (**h**) The amount of mitochondrial HKII as ratio to VDAC normalized to WT. (**i**) The amount of cytosolic HKII as ratio to tubulin normalized to wt. n = 6 per group. Data are shown as mean + SEM. Differences between groups were analysed using Mann-Whiney U test *p < 0.05.

Baseline levels of LDH and HK were determined in hearts of mice of protocol 1 (Fig. 2a). Hearts of CypD^−/−^ mice contained significantly more LDH compared to control (2.0 ± 0.2 vs 1.3 ± 0.07 U/mg protein respectively; p = 0.009). The total amount of HK activity and mitochondrial CS activity did not differ between both groups (Fig. 2b and c).

At baseline no differences were observed between WT and CypD^−/−^ hearts on EDP, DLVP, heart rate, RPP and flow (Table 1).

**Table 1:** Baseline characteristics I/R experiments.

	Glucose only				Glucose, lactate, pyruvate, L-glutamine			
	Wt con	Wt IPC	CypD^−/−^ con	CypD^−/−^ IPC	Wt con	Wt IPC	CypD^−/−^ con	CypD^−/−^ IPC
EDP (mmHg)	3.8 ± 0.4	3.9 ± 0.4	5.0 ± 1.1	7.2 ± 1.4	5.8 ± 1.3	4.7 ± 0.8	5.0 ± 0.5	6.0 ± 0.6
DLVP (mmHg)	115.8 ± 3.4	106.1 ± 4.6	114.7 ± 7.6	103.6 ± 8.4	110.1 ± 8.0	108 ± 3.7	106.1 ± 6.7	100.7 ± 7.2
HR (bpm)	411 ± 14	383 ± 17	425 ± 11	410 ± 11	394 ± 17	387 ± 19	400 ± 20	390 ± 17
RPP (HR*DLVP)	47819 ± 2548	40321 ± 2192	48710 ± 3258	41995 ± 2587	43651 ± 3825	41658 ± 2258	41995 ± 2586	39171 ± 3079
Flow (ml/min/g HW)	12.3 ± 0.6	11.4 ± 0.5	11.6 ± 0.5	15.1 ± 1.8	14.1 ± 2.0	11.6 ± 0.8	11.6 ± 1.1	11.3 ± 1.1

EDP: end diastolic pressure; DLVP: Developed left-ventricular pressure; HR: Heart rate; RPP: Rate pressure product; HW: heart weight. Data are presented as mean ± SEM, 6–13 animals per group. Mann-Whitney U test has been performed to test between groups.

### CypD ablation does not alter normoxic mtHKII

When examining the mitochondrial and cytosolic fraction separately no difference was observed in HK activity between WT and CypD^−/−^ mice (Fig. 2d and e). The semi-quantitative amount of HKII in these compartments was determined using Western blot. Remarkably, although others have shown that CypD affects HKII binding to mitochondria in non-cardiac cells^20,33^, we observed similar binding of HKII to mitochondria in WT and CypD^−/−^ hearts (Fig. 2f and h). Also no differences were observed in the amount of cytosolic HKII (Fig. 2g and i).

### CypD ablation does not alter end-ischemic mtHKII and is not associated with loss of IPC in glucose-only perfused heart

**Figure 3:**
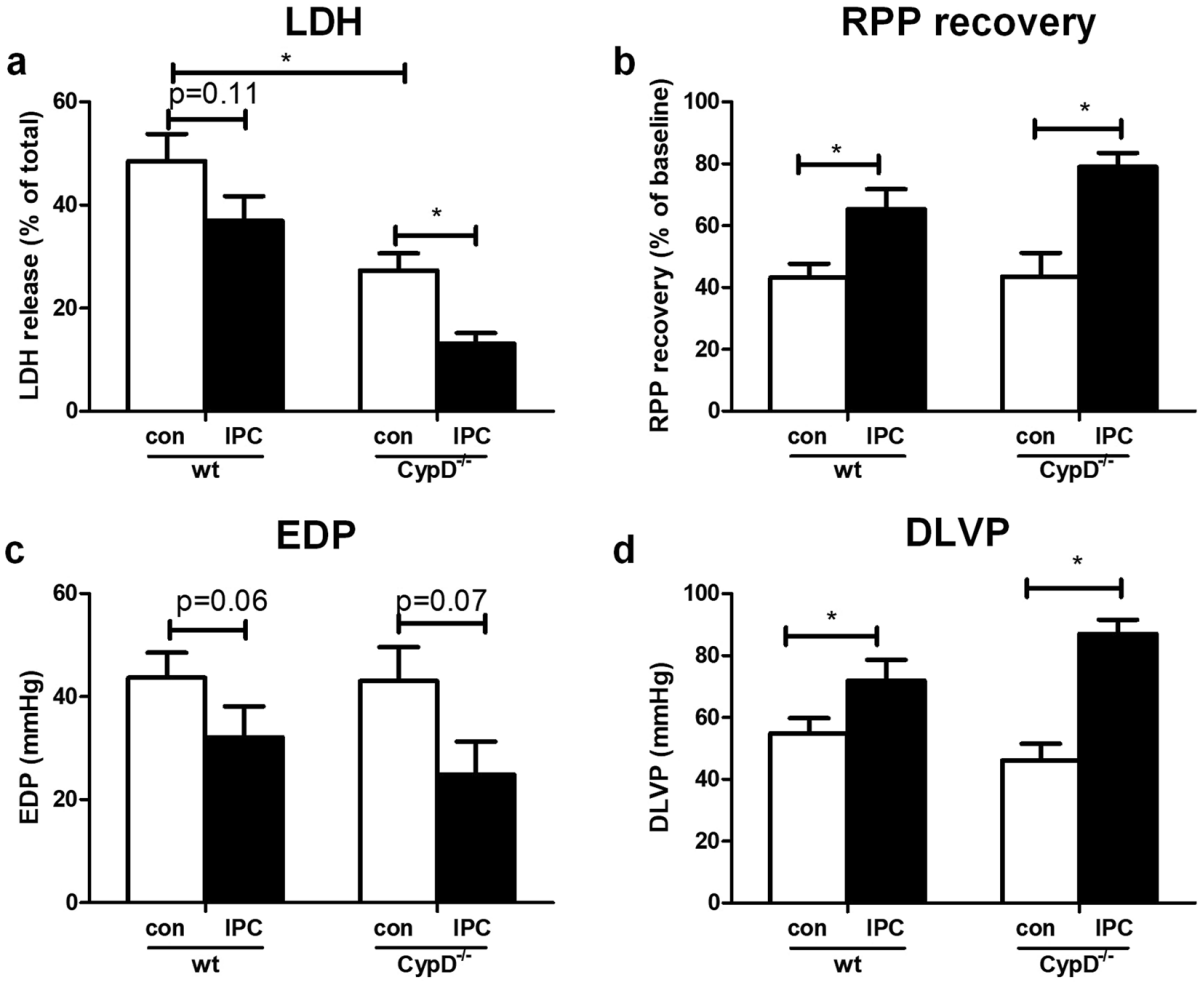
CypD ablation decreases I/R injury and CypD^−/−^ hearts are protected by IPC in hearts perfused with glucose only. (**a**) Lactate dehydrogenase (LDH) release during reperfusion as a percentage of total LDH in the heart. (**b**) Rate pressure product (RPP) at the end of reperfusion as percentage of baseline values. (**c**) End-diastolic pressure (EDP) at the end of reperfusion. (**d**) Developed left ventricular pressure (DLVP) at the end of reperfusion. N = 7–13 per group. Data are shown as mean + SEM. Mann-Whitney U tests were performed to test for significance between 1) control treated WT and CypD^−/−^ animals 2) control and IPC treated animals in WT and 3) CypD^−/−^ animals. *p < 0.05.

Twenty-five minutes ischemia plus 45 min reperfusion resulted in 49 ± 5% cell death (total loss of LDH) and 43 ± 5% RPP recovery with an EDP of 44 ± 5 mmHg and a DLVP of 55 ± 5 mmHg in WT hearts (Fig. 3). Deletion of CypD reduced I/R-induced cell death by 42% (p = 0.008) to 27 ± 3% cell death, but was without effect on cardiac contracture, RPP recovery and DLVP at the end of reperfusion when compared to WT.

IPC was only marginally effective in WT animals, resulting in a significant improvement in RPP recovery to 65 ± 6% (p = 0.01) and DLVP to 72 ± 7 mmHg (p = 0.04), but only a non-significant improvement for LDH release to 37 ± 5% (p = 0.1) or contracture development to 32 ± 6 mmHg (p = 0.06). Remarkably, in contrast to what is reported in literature^5,7^, IPC was markedly effective in CypD^−/−^ hearts. IPC reduced cardiac injury in CypD^−/−^ hearts, as reflected by a decreased LDH release (27 ± 3% to 13 ± 2% of total LDH; p = 0.007), improved RPP recovery (44 ± 8% to 79 ± 4%; p = 0.01), diminished cardiac contracture (43 ± 7 to 25 ± 6 mmHg; p = 0.07) and improved DLVP (46 ± 5 to 87 ± 5 mmHg; p = 0.001) at the end of reperfusion.

**Figure 4:**
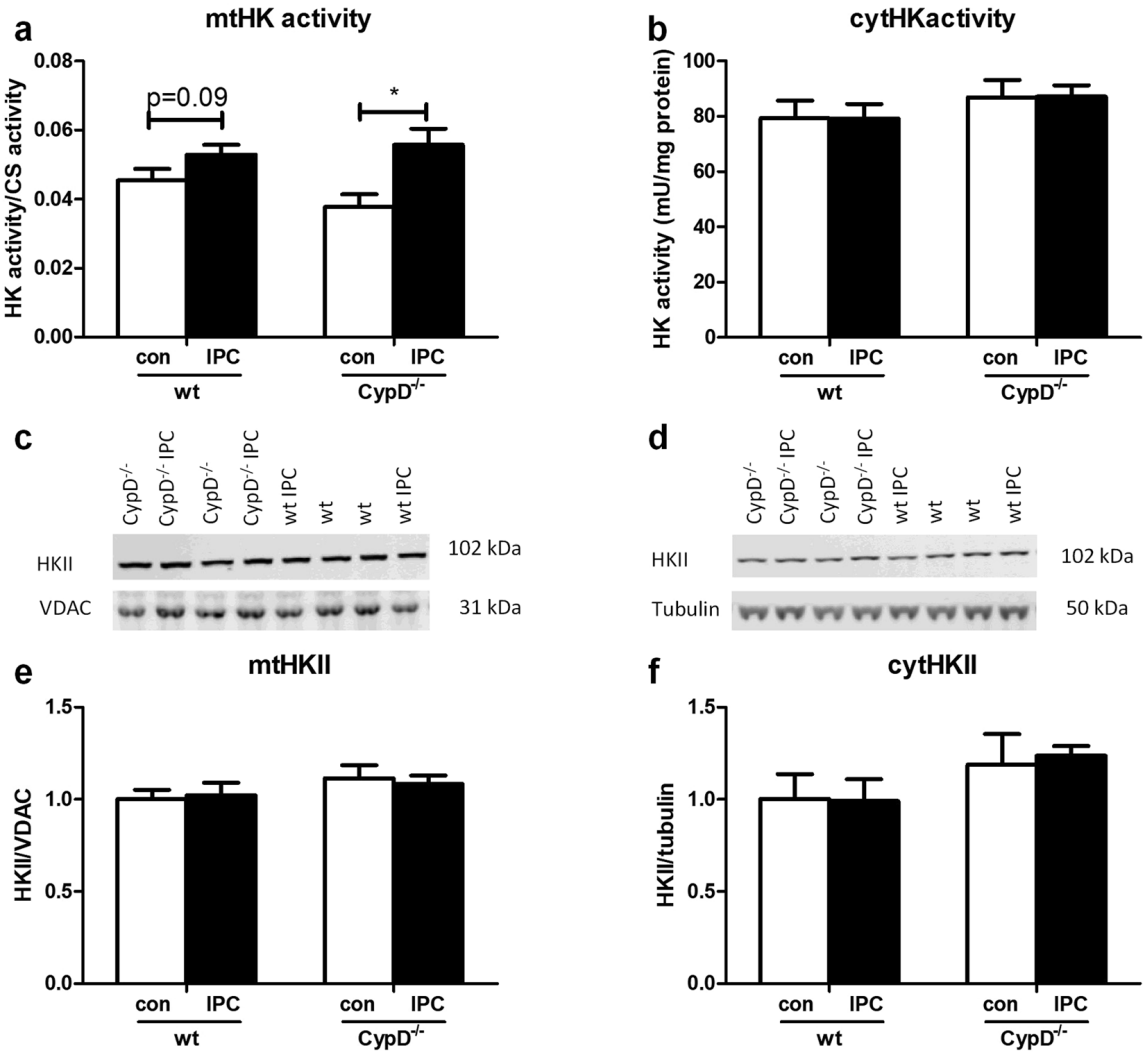
CypD and IPC have no effect on end-ischemic mtHKII in hearts perfused with glucose only. (**a**) End-ischemic mitochondrial HK activity as ratio to CS activity. (**b**) Cytosolic HK activity normalized to protein content. (**c**) Representative Western blot images of mitochondrial HKII/VDAC and (**d**) cytosolic HKII/tubulin. The amount of end-ischemic mitochondrial (**e**) and cytosolic (**f**) HKII as ratio of VDAC and tubulin respectively, normalized to WT control. n = 6 per group. Data are shown as mean + SEM. Mann-Whitney U tests were performed to test for significance between 1) control treated WT and CypD^−/−^ animals 2) control and IPC treated animals in WT and 3) CypD^−/−^ animals. p < 0.05.

Next, we examined in a different series of experiments whether CypD and IPC effects on cardiac I/R injury were mirrored by end-ischemic mtHK (Fig. 4). CypD ablation did not alter end-ischemic mtHK activity (Fig. 4a) or mtHKII amount (Fig. 4c) as compared to WT. Similar results were observed for end-ischemic cytosolic HK activity or cytosolic HKII presence (Fig. 4b and d, respectively). In contrast, the strong cardioprotective effect of IPC in CypD^−/−^ hearts was associated with significant increased mtHK activity, although this increased HK activity could not be further allocated to increased mtHKII or decreased cytosolic HKII amount using the WB technique. IPC in WT hearts only caused a non-significant increase in mtHK activity from 0.045 ± 0.003 to 0.052 ± 0.003 (p = 0.09), without any effect on other indices of HK cellular redistribution

These data clearly indicates that CypD ablation effects on cardiac I/R are independent of mtHK alterations in glucose-only perfused mouse hearts.

### IPC effects on time of contracture depends on substrate availability in isolated mouse heart

**Figure 5:**
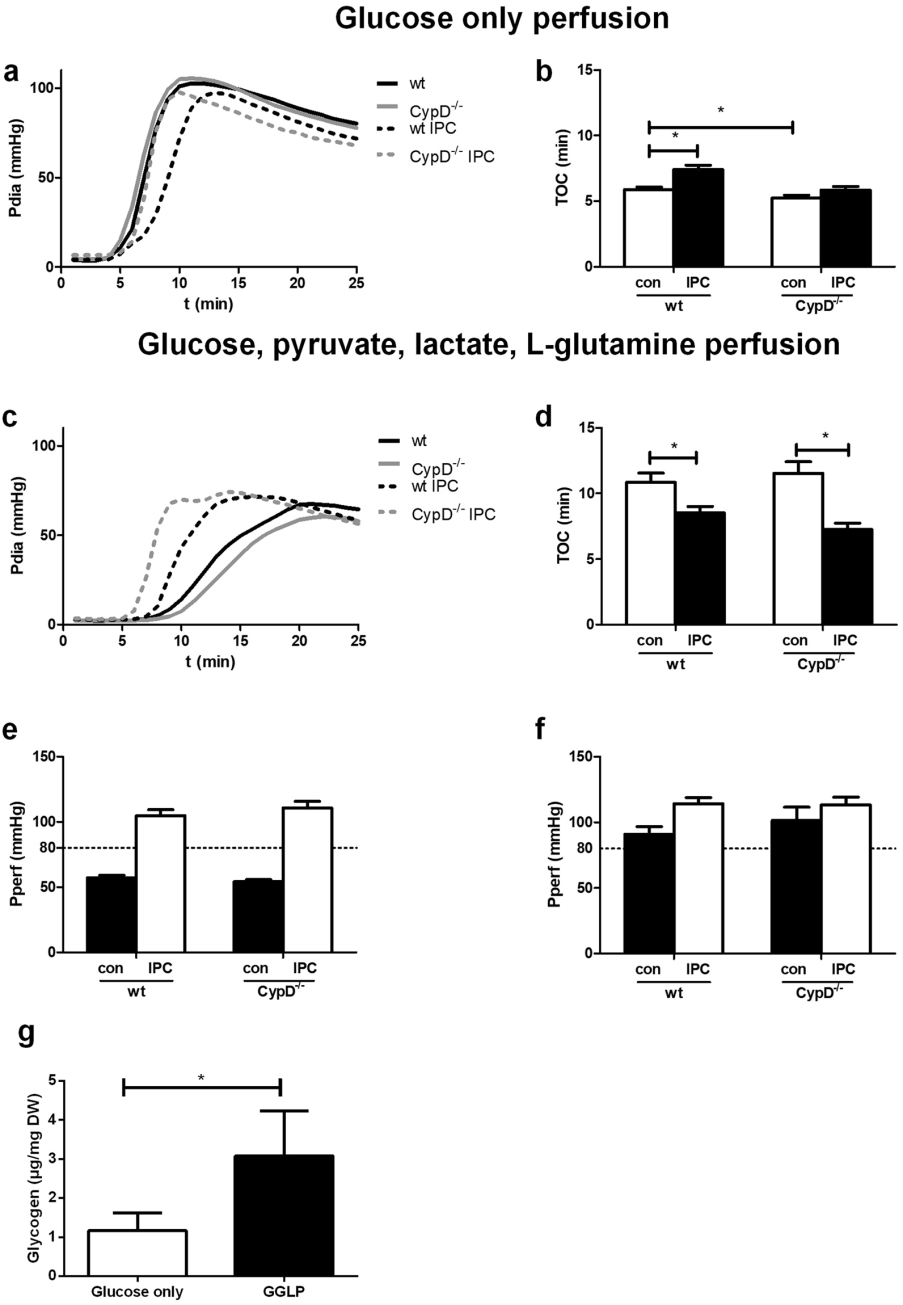
Metabolic substrates influence time of contracture during ischemia. Diastolic pressure (**a** + **c**) and time of contracture (**b** + **d**) during ischemia and perfusion pressure (Pperf) at the end of baseline (**e** + **f**) in the presence of glucose only (**a** + **b** + **e**) or glucose, pyruvate, lactate and L-glutamine (**c** + **d** + **f**). (**g**) Glycogen content in the heart after 30 min basal perfusion with the different perfusates. n = 12–19 (**a**–**f**) and n = 6–7 (**g**) per group. Data are shown as mean + SEM. For TOC Mann-Whitney U tests were performed to test for significance between 1) control treated WT and CypD^−/−^ animals 2) control and IPC treated animals in WT and 3) CypD^−/−^ animals. Glycogen data weas analysed using an Independent T-test. *p < 0.05.

The protective effect of IPC on I/R injury was only marginally present. Glycogen depletion is one of the factors contributing to IPC^34,35^. Time to onset of contracture (TOC) is a marker of glycogen depletion^36^ and IPC is also commonly associated with an earlier TOC due to glycogen depletion during the short ischemic periods of the IPC protocol^34^. In our glucose-only perfused mouse hearts, however, no smaller TOC was observed after IPC (Fig. 5a and b). On the contrary, TOC was significantly smaller in WT control animals compared to WT IPC animals (5.9 ± 0.2 vs 7.4 ± 0.3 min; p < 0.001, respectively). This early TOC, indirectly suggesting extensive glycogen depletion, might be explained by that 11 mM glucose provides insufficient substrate to prevent glycogen depletion under normoxic conditions. Therefore, similar experiments were performed in the presence of not only glucose, but also of physiological concentrations of glutamine, lactate and pyruvate, substrates known to be highly extracted and used by the heart^37^. Indeed, in the presence of these substrates TOC increased significantly from 5.9 ± 0.2 to 10.8 ± 0.7 min (p < 0.001) in WT control animals, indicating sparing of glycogen during normoxic perfusion. In addition, in both WT and CypD^−/−^ animals IPC now significantly reduced TOC (from 10.8 ± 0.7 to 8.5 ± 0.5 min in WT and from 11.5 ± 0.9 to 7.3 ± 0.5 min in CypD^−/−^ animals) (Fig. 5c and d). Furthermore, we also noticed that with the additional substrates the isolated mouse heart preparation was more stable in terms of constancy of perfusion pressure in this constant flow model (Fig. 4e and f). To verify that glucose only perfusion depleted glycogen, glycogen content of the heart was measure after 30 min basal perfusion with both perfusion conditions. Indeed, glycogen was 2.6 times higher in hearts perfused with glutamine, lactate and pyruvate in addition to glucose (1.17 ± 0.18 vs 3.08 ± 0.43 µg/mg dry weight; p = 0.003) (Fig. 4g). The different perfusion conditions did not show any differences in cardiac energetics. PCr, ATP, PCr/ATP ratio, a clinical indicator for the energy state of the heart, Pi, Cr or ΔG_ATP_ were comparable between the conditions (Table 2). Even though cardiac energetics were not altered, these data still indicate that for the isolated mouse heart generating left ventricular pressure of more than 90 mmHg, glucose only perfusions are insufficient.

**Table 2:** Cardiac energetics.

	Glucose-only	GGLP	P
ATP (µg/g DW)	25.1 ± 1.0	25.7 ± 1.2	0.69
PCr (µg/g DW)	21.8 ± 1.9	24.8 ± 2.1	0.32
Cr (µg/g DW)	31.3 ± 1.8	30.2 ± 2.7	0.75
Pi (µg/g DW)	16.5 ± 1.3	12.9 ± 2.6	0.25
PCr/ATP	0.87 ± 0.08	0.96 ± 0.07	0.43
ΔG_ATP_ (kJ)	50.5 ± 0.4	49.3 ± 0.7	0.22

GGLP: glucose, L-glutamine, lactate, pyruvate; PCr: phosphocreatine; Cr: Creatine; Pi: inorganic phosphate; ΔG_ATP_: free energy of ATP. Data are presented as mean ± SEM, 6–7 animals per group. An independent T- test has been performed to test between groups.

Next, we examined whether the now observed reduction in TOC with IPC translated in a more prominent increase of mitochondrial HK with IPC as compared to no-IPC in both WT and CypD^−/−^ hearts, as has been suggested by Pasdois *et al*.^19^.

### CypD ablation does increase end-ischemic mtHK activity in mixed substrate-perfused heart

Cardiac mechanical performance and flow at baseline conditions were similar between WT and CypD^−/−^ isolated mouse hearts using the mixed-substrate KHB (Table 1).

**Figure 6:**
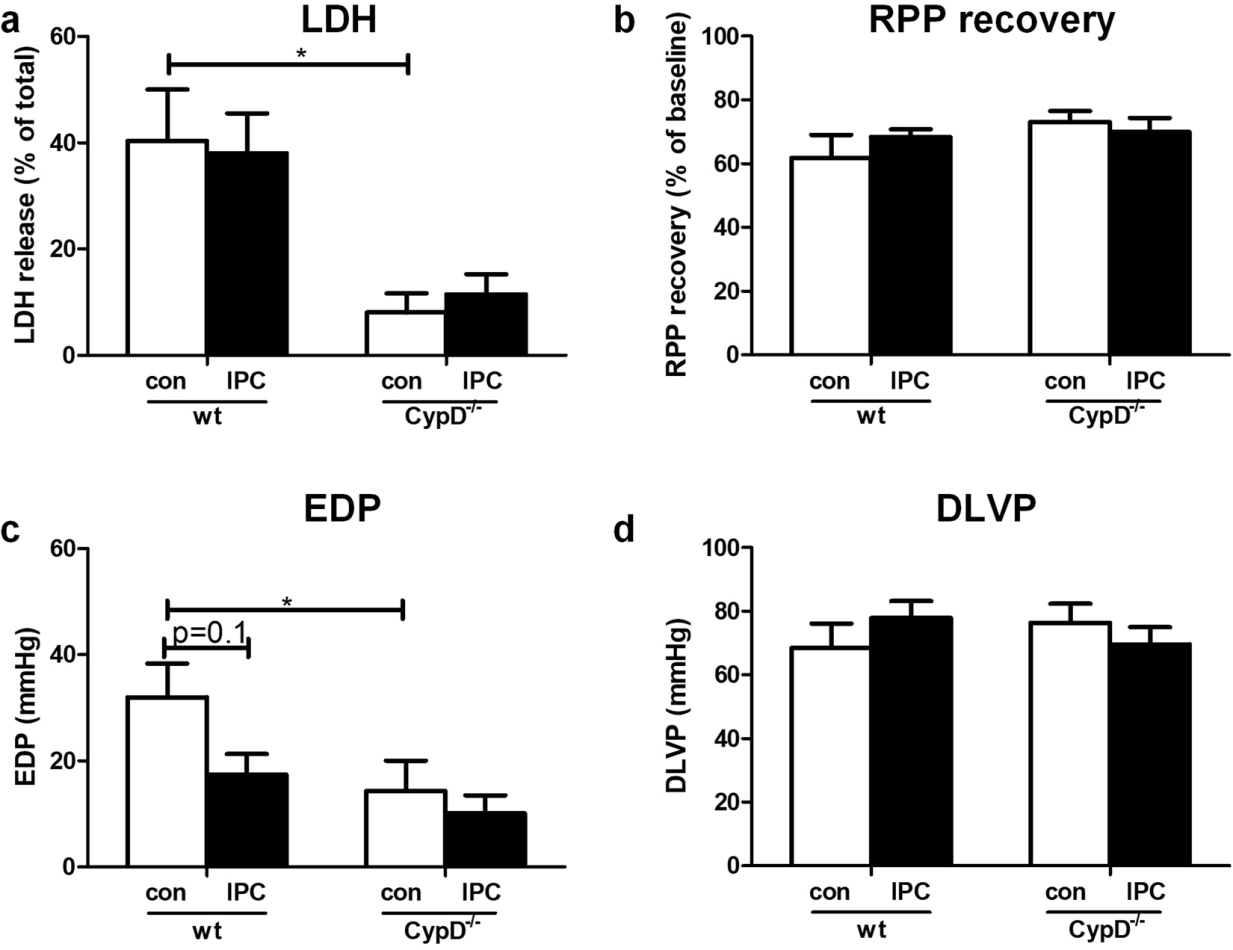
CypD ablation reduces I/R injury in hearts perfused with glucose, pyruvate, lactate and glutamine. (**a**) Lactate dehydrogenase (LDH) release during reperfusion as a percentage of total LDH in the heart. (**b**) Rate pressure product (RPP) at the end of reperfusion as percentage of baseline values. (**c**) End-diastolic pressure (EDP) at the end of reperfusion. (**d**) Developed left ventricular pressure (DLVP) at the end of reperfusion. n = 6–7 per group. Data are shown as mean + SEM. Mann-Whitney U tests were performed to test for significance between 1) control treated WT and CypD^−/−^ animals 2) control and IPC treated animals in WT and 3) CypD^−/−^ animals. *p < 0.05.

Under these perfusion conditions, cell death in WT hearts at the end of reperfusion was 40 ± 10%, with a RPP recovery of 62 ± 7%, an EDP of 32 ± 6 mmHg and a DLVP of 69 ± 8 mmHg (Fig. 6). Surprisingly, the cardioprotective effects of CypD deletion on I/R injury were increased as compared to glucose-only perfused hearts. Deletion of CypD reduced I/R-induced cell death by 79%, as reflected by the lower LDH release (8.1 ± 4%; p = 0.002), and now significantly improved recovery of cardiac function, shown by less cardiac contracture development (EDP: 14 ± 6 mmHg; p = 0.04) (Fig. 6). LDH content remaining in heart, provided similar (reciprocal) results as the LDH measurement in effluent, suggesting that LDH release is a proper measure of irreversible IR damage in our isolated mouse model (data not shown).

Despite the observation that IPC in the mixed substrate-perfused hearts now induced a shorter TOC, indicating less glycogen breakdown during ischemia in IPC-treated heart, overall IPC protective effects were actually diminished. IPC reduced cardiac contracture in WT animals from 32 ± 6 to 17 ± 2 mmHg (p = 0.1), but was without effect on RPP recovery and LDH activity. In contrast to what we observed when hearts were perfused with glucose only, IPC was now without effects on LDH release (8.1 ± 3.6 vs 11.5 ± 3.8%; p = 0.2), RPP recovery (73.1 ± 3.5 vs 70.0 ± 4.4%; p = 0.5) and cardiac contracture (14.3 ± 5.7 vs 10.1 ± 3.4 mmHg; p = 0.5) in CypD^−/−^ hearts (Fig. 6).

**Figure 7:**
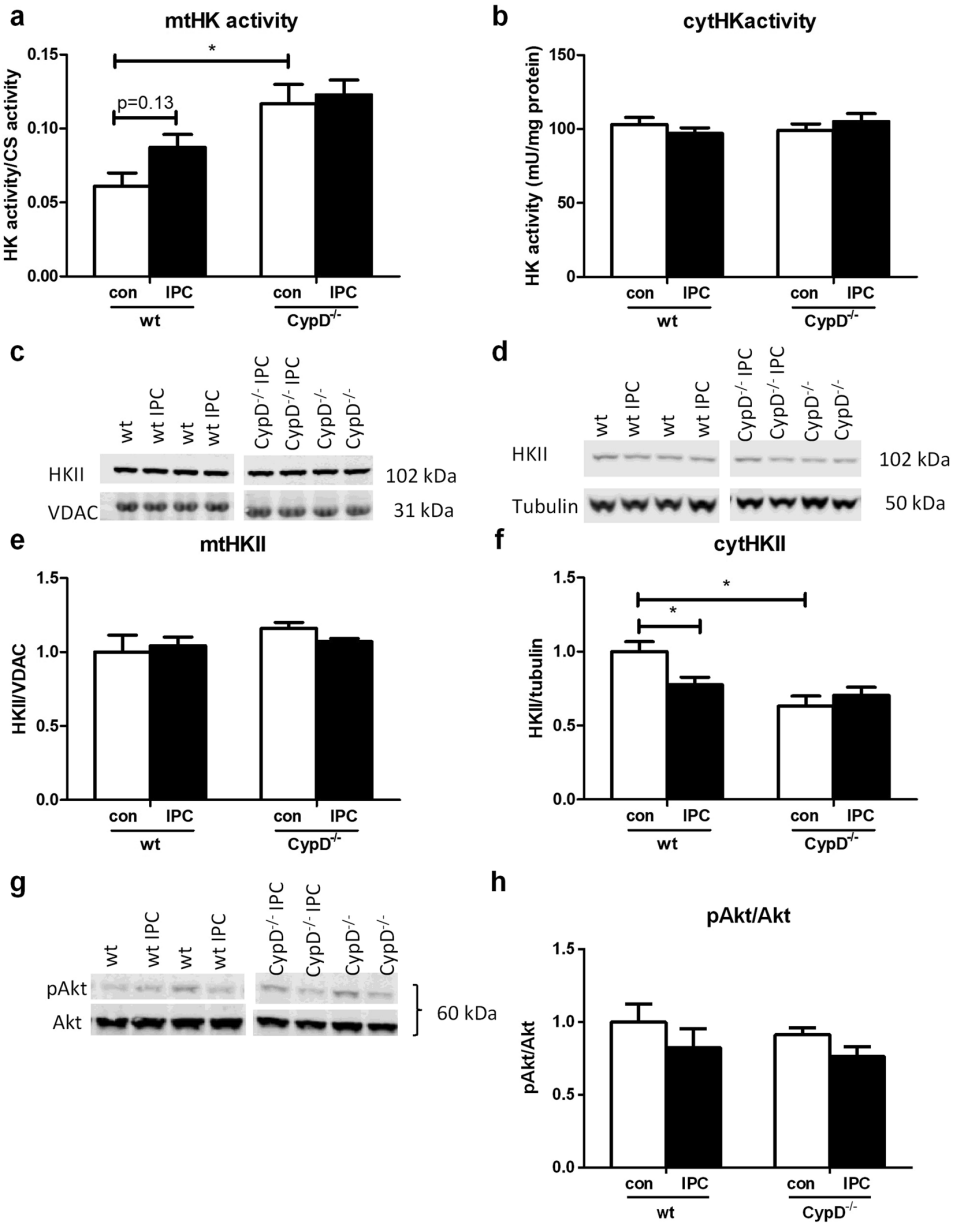
CypD and IPC have no effect on end-ischemic mtHKII in hearts perfused with glucose, pyruvate, lactate and glutamine. (**a**) End-ischemic mitochondrial HK activity as ratio to CS activity. (**b**) Cytosolic HK activity normalized to protein content. (**c**) Representative Western blot images of mitochondrial HKII/VDAC and (**d**) cytosolic HKII/tubulin. The amount of end-ischemic mitochondrial (**e**) and cytosolic (**f**) HKII as ratio of VDAC and tubulin respectively, normalized to WT control. (**g**) Representative Western blot image and quantification (**h**) of whole homogenate pAkt/Akt, normalized to WT control. n = 6 per group. Data are shown as mean + SEM. Mann-Whitney U tests were performed to test for significance between 1) control treated WT and CypD^−/−^ animals 2) control and IPC treated animals in WT and 3) CypD^−/−^ animals. *p < 0.05.

The improved protection against I/R injury with CypD ablation was associated with significantly increased mtHK activity (0.061 ± 0.009 vs 0.12 ± 0.01; p = 0.02) and reduced cytosolic HKII amount (1 ± 0.07 vs 0.63 ± 0.07; p = 0.004) at end-ischemia (Fig. 7a,d and f, respectively). No effects of CypD ablation were observed on mtHKII amount or cytosolic HK activity (Fig. 7c,e and b, respectively). IPC significantly reduced cytosolic HKII in WT animals from to 0.77 ± 0.05 (p = 0.02), with a non-significant trend for increased mtHK activity to 0.087 ± 0.009 (p = 0.2). The absence of a protective effect of IPC on I/R injury was mirrored by the observation that IPC was now without an effect on any of the HK determinations in CypD^−/−^ hearts. Differences in end-ischemic HK were not associated with differences in end-ischemic Akt phosphorylation (Fig. 7g and f). There were no differences between pAkt/Akt between wt and CypD^−/−^ hearts (1.0 ± 0.12 vs 0.91 ± 0.05; p = 0.9). pAkt/Akt was non-significantly decreased after IPC in both groups (1.0 ± 0.12 vs 0.82 ± 0.12; p = 0.3 in the WT and 0.91 ± 0.05 vs 0.76 ± 0.07; p = 0.07 in the CypD^−/−^ hearts).

## Discussion

In this study we observed that 1) CypD is not necessary for HKII to bind to mitochondria in the heart, 2) deletion of the mitochondrial CypD enzyme augments hexokinase activity at the mitochondria during cardiac ischemia in the all substrates series, 3) CypD is not always mandatory for IPC protective effects, 4) glucose as sole metabolic substrate is insufficient for the Langendorff perfused mouse

Multiple studies in cancer cells have shown that CypD inactivation leads to release of HKII from the mitochondria, assuming CypD activity is necessary for HKII binding to the mitochondria^20,33^. As both the presence of mtHKII and the absence of CypD protect the heart against I/R injury^5–10,13,14,16,19^, we studied in intact hearts whether HKII binding to mitochondria was dependent on the presence of CypD. In our study however, we found under baseline, non-ischemic, conditions that in CypD^−/−^ hearts HKII was bound to mitochondria and that the amount of mtHKII binding or mtHK activity did not differ between WT and CypD^−/−^ hearts. The difference between this study and other studies might be caused by difference in cell type. Above mentioned studies were all performed in cancer cells, where the amount of mtHKII is higher and significant changes in the activation status of cellular signalling pathways are present. In addition, none of these studies have been performed in CypD^−/−^ cells, but rather using CsA. In CypD^−/−^ animals, compensatory mechanisms might have restored mtHKII to WT levels. Conflicting results on the effects of proteins on I/R have been reported for other proteins, eg p66Hsc^38^. Also for P66Shc effects on I/R are critically dependent on the specifics of the experimental model. Summarised, during baseline, non-ischemic, conditions, chronic CypD deletion is without effect on mtHKII in the heart.

As in other studies^5,7^ we observed a protective effect of CypD^−/−^ on I/R injury. As a novel observation, the present study demonstrates that this CypD^−/−^ protective effect was more pronounced in the presence of glucose, L-glutamine, lactate and pyruvate than with glucose only. In the presence of multiple substrates, the protective effect was associated with an increase in end-ischemic mtHK activity, without changes in the amount of mtHKII or cytosolic HK activity. This higher activity could have been caused by HKI translocation, since hexokinase activity is the activity of both HKI and HKII. Although we cannot exclude a translocation of HKI, in other studies we have shown that IPC can increase the amount of HKII, but has no effect on HKI^15^. In addition, all articles that have examined HK translocation in the heart have ascertained that HKI does not translocate with any intervention^13,15,39^. Furthermore, if HKI was translocated we would have expected a decrease in cytosolic HK activity which was not observed. Therefore, we hypothesize that the higher hexokinase activity was not caused by HK translocation, but by increased activity due to post-translational modification. The activation of different kinases, like Akt and AMPK, has been shown to be able to phosphorylate hexokinase and increase its activity^10,40,41^ Therefore we hypothesized that an increase in pAkt caused the increase in HK activity. In contrast to our hypothesis, CypD^−/−^ did not increase end-ischemic pAkt. Nor was end-ischemic pAkt increased after IPC. Others did also not observe increased pAkt in the hearts of CypD^−/−^ mice^5^. Therefore, further studies are necessary to examine how CypD^−/−^ increases end-ischemic hexokinase activity.

Summarized, during conditions of ischemia, CypD deletion is associated with increased mtHK activity in the heart.

We observed that CypD^−/−^ hearts could still be preconditioned using IPC when perfused with glucose only. The absence of a preconditioning effect in CypD^−/−^ hearts in the mixed substrate perfusion might be caused by the already good recovery in the control group. Cell death was already decreased to 8% in CypD^−/−^ control hearts. Protection of CypD^−/−^ hearts using IPC indicates that other mechanisms for IPC protection than delayed opening of the MPTP are operative in the heart. Ruiz-Meana *et al*.^9^ showed that an important part of necrosis after short periods of ischemia was caused by reperfusion contracture that mechanically resulted in sarcolemmal rupture. These mechanisms were independent of CypD and the MPTP. IPC could affect these causes of cell death and thereby improve cardiac function and survival after I/R also in CypD^−/−^ animals. The observation that CypD^−/−^ hearts in this study could be preconditioned is in contrast to findings by the group of Yellon^5,7^. This difference might be caused by the difference in model employed. We used a Langendorff perfused mouse heart while the other studies were performed *in vivo* or in cardiomyocytes.

In this study we observed only a marginal effect of IPC in WT animals in the presence of glucose only and no increase in mtHK in IPC hearts. Pasdois *et al*.^19^ showed that there is a link between cardioprotection, mtHK and pre-ischemic glycogen content. A lower glycogen content of the heart, which is one of the consequences of IPC, is associated with a better outcome after I/R^34,35,42^. A smaller TOC is a sign of early inhibition of glycolysis due to depleted glycogen. This decreases glucose-6-phosphate and reduces the drop in pH, which in turn reduces the amount of HKII released from mitochondria^19^. In our hearts perfused with glucose only, no reduction of TOC was observed in IPC animals. On the contrary, TOC was significantly smaller in WT control animals compared to WT IPC animals. This might have been caused by IPC slowing down energy metabolism during ischemia^43^. We hypothesized that the absence of a reduction in TOC after IPC was caused by extensive glycogen depletion during normoxic perfusion, because the 11 mM glucose, used in our model, provided insufficient substrate. In the absence of glycogen, the IPC-induced slowing down of metabolism might have overridden the IPC effects of glycogen, and increased TOC. Therefore experiments were repeated in the presence of glucose, glutamine, pyruvate and lactate as substrates for the heart. We chose these substrates, because we think that it 1) mimics the *in vivo* physiological conditions more closely, and thereby improves translatability of the isolated heart model and 2) provides a more stable preparation. Indeed, with this protocol glycogen concentrations were significantly higher and TOC was now significantly smaller in preconditioned hearts compared to control hearts. However, still no increase in mtHKII after IPC or protective effect of IPC could be observed. So glycogen depletion cannot explain the absence of an increase in mtHKII and preconditioning in the WT animals. Others have also shown that glycogen depletion is not the only determinant for the effectiveness of IPC^42,44^. We have recently shown that using this mixed substrate perfusate will result in rather low glycolysis rates^45^. Knowing that increases in glycolysis may partly underlie cardioprotection and alterations in mitochondrial function following IPC^27,46,47^, the impairment of glycolysis using the mixed substrate perfusate may be responsible for the diminished IPC potential with the mixed substrate perfusate. Further studies are necessary to elucidate this with more certainty.

In the Langendorff perfused rat heart it has been shown that glucose has only a small contribution to acetyl-CoA formation in the absence of insulin^37^. Indeed, we observed a small TOC and a significantly lower amount of glycogen in control hearts with glucose-only perfusion indicating that glucose as sole metabolic substrate is insufficient for the Langendorff perfused mouse heart and the heart therefore is depleting its glycogen storage^36^. Lactate and pyruvate have a significant contribution to acetyl-CoA formation, especially in the absence of insulin^37^, which can explain the delayed TOC in the presence of these substrates. In addition, glutamine is the most abundant amino acid that can be found in the blood and amino acids are important players in cardiac metabolism and IR studies^48,49^. Therefore we believe it is important to have glutamine present in studies examining I/R, knowing that also I/R is largely metabolically driven.

Another indication that glucose as singular substrate is insufficient in the Langendorff perfused mouse heart is the decrease in perfusion pressure we observed in glucose-only perfused hearts, but not in hearts perfused with additional substrates.

From our data we summarised that only providing glucose as substrate to the isolated mouse heart results in an unstable heart preparation, and should be avoided in future experiments.

It should be taken into account that this study also has several limitations. One of the limitations is the use of an *ex vivo* instead of *in vivo* model, because it cannot recapitulate the complexity of the *in vivo* setting^50,51^. However, it was necessary for the quick determination of the labile binding of HKII to mitochondria, without contamination with other tissue and/or blood. In addition, the isolated heart also allowed to study contracture development during ischemia, which was an important parameter in the present study. Finally, cardiac mechanical performance that can totally be ascribed to the functioning of the heart per se, and not to systemic pre-afterload, or changes in the hormonal/neural environment of the heart in the *in vivo* condition, allowed improved characterization of how CypD deletion in the heart directly affected cardiac mechanical performance following I/R, which is one of the main topics of the current manuscript.

Secondly, the use of LDH as determinant of cardiac necrosis, instead of the golden standards TTC or histology^50^ may be a limitation. As with TTC staining, cell death measured by LDH release depends on the wash-out of dehydrogenases, which might be obstructed. Therefore, we have also measured LDH in the heart at the end of reperfusion. This correlated well with the LDH release data in effluent. In addition, LDH has previously been shown to correlate well with infarct size as measured by TTC staining (e.g.^29–31^).

Another limitation of this study is the use of a high, non-physiological, concentration of glucose (11 mM), even in the more physiological multi-substrate perfusate. A high concentration of glucose is necessary to facilitate glucose uptake in the cardiomyocyte, since no insulin was present. This mild hyperglycemia, although being a common and necessary characteristic of the Langendorff perfused mouse heart model^52,53^, might have affected our results^54^.

In conclusion, in contrast to cancer cells, our results show that HK can still bind to mitochondria in the absence of CypD in the heart, and that mtHK may be involved in reduction of I/R injury by CypD ablation.

### Data availability

The datasets generated during and/or analysed during the current study are available from the corresponding author on reasonable request.
